# Measurements of Atmosphere–Biosphere Exchange of Oxidized Nitrogen and Implications for the Chemistry of Atmospheric NO_*x*_

**DOI:** 10.1021/acs.accounts.3c00090

**Published:** 2023-06-22

**Authors:** Erin R. Delaria, Ronald C. Cohen

**Affiliations:** † Atmospheric Chemistry and Dynamics Laboratory, NASA Goddard Space Flight Center, Greenbelt, Maryland 20771, United States; ‡ Oak Ridge Associated Universities, Oak Ridge, Tennessee 37830, United States; ¶ Department of Chemistry, University of California, Berkeley, Berkeley, California 94720, United States; § Department of Earth and Planetary Science, University of California, Berkeley, Berkeley, California 94720, United States

## Abstract

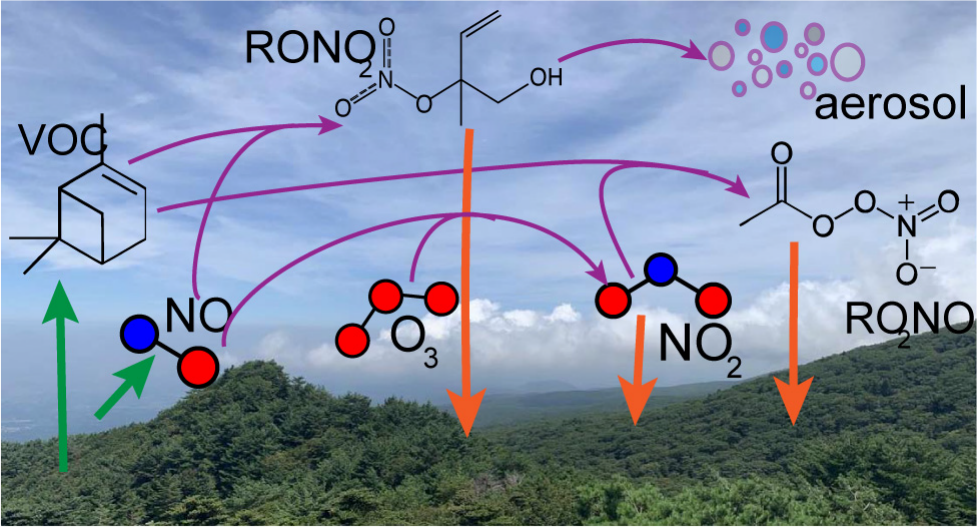

The atmosphere–biosphere exchange of nitrogen oxides plays a key role in determining the composition of reactive nitrogen in terrestrial vegetated environments. The emission of nitric oxide (NO) from soils is an important atmospheric source of reactive nitrogen. NO is rapidly interconverted with NO_2_, making up the chemical family NO_*x*_ (NO_*x*_ ≡ NO_2_ + NO). NO_*x*_ further reacts with the oxidation products of volatile organic compounds (VOCs) to form the functionalized nitrogen oxide groups acyl peroxynitrates (APNs = R(O)O_2_NO_2_) and alkyl nitrates (ANs = RONO_2_). Both canopy-level field measurements and laboratory studies suggest that the absorption of nitrogen dioxide NO_2_ and APNs by vegetation is a significant sink of atmospheric NO_*x*_, removing a large fraction of global soil-emitted NO_*x*_ and providing key control on the amounts and lifetimes of NO_*x*_ and reactive nitrogen in the atmosphere. Nitrogen oxides influence the production of surface O_3_ and secondary aerosols. The balance of the emission and uptake of nitrogen oxides thus provides a mechanism for the regulation of regional air quality. The biosphere, via this biogeochemical cycling of nitrogen oxides, is becoming an increasingly important determining factor for airborne pollutants as much of the world continues to reduce the amount of combustion-related nitrogen oxide emissions. Understanding the function of the biosphere as a source and sink of reactive nitrogen is therefore ever more critical in evaluating the effects of future and current emissions of nitrogen oxides on human and ecosystem health.

Laboratory measurements of the foliar deposition of NO_2_ and other reactive nitrogen species suggest that there is a substantial diversity of uptake rates under varying environmental conditions and for different species of vegetation that is not currently reflected in the widely utilized chemical transport models. Our branch chamber measurements on a wide variety of North American tree species highlight the variability in the rates of both photosynthesis and nitrogen oxide deposition among several different nitrogen oxide compounds. Box-modeling and satellite measurement approaches demonstrate how disparities between our understanding of nitrogen oxide foliar exchange in the laboratory and what is represented in models can lead to misrepresentations of the net ecosystem exchange of nitrogen. This has important implications for assumptions of in-canopy chemistry, soil emissions of NO, canopy reductions of NO_*x*_, lifetimes of trace gases, and the impact of the biosphere on air quality.

## Key References

- DelariaE. R.; VieiraM.; CremieuxJ.; CohenR. C.Measurements of NO and NO_2_ exchange between the atmosphere and Quercus agrifolia. Atmospheric Chemistry and Physics2018, 18, 14161–1417310.5194/acp-18-14161-2018.^1^*Laboratory branch measurements using the single dynamic chamber method showed substantial uptake of NO_2_ by California live oak trees, comparatively slow NO deposition, and no evidence for an NO_2_ compensation point.*
- DelariaE. R.; PlaceB. K.; LiuA. X.; CohenR. C.Laboratory measurements of stomatal NO_2_ deposition to native California trees and the role of forests in the NO_*x*_ cycle. Atmospheric Chemistry and Physics2020, 20, 14023–1404110.5194/acp-20-14023-2020.^2^*Chamber measurements on 10 different tree species demonstrate variability in the NO_2_ deposition rates that was driven primarily by the variability in the stomatal conductance, regardless of soil nitrogen or drought status.*
- PlaceB. K.; DelariaE. R.; LiuA. X.; CohenR. C.Leaf Stomatal Control over Acyl Peroxynitrate Dry Deposition to Trees. ACS Earth and Space Chemistry2020, 4, 2162–217010.1021/acsearthspacechem.0c00152.^3^*Laboratory chamber measurements demonstrate variability in PAN stomatal deposition rates between tree species, show negligible deposition to leaf cuticles, and provide evidence that reactions within the leaf’s mesophyll slow PAN stomatal uptake*.

## 1 Introduction

The chemistry of atmospheric NO_*x*_ (NO_*x*_ ≡ NO + NO_2_) leads to the formation of both toxic and phytotoxic atmospheric products, including O_3_ and secondary aerosols. NO_*x*_ chemistry also controls the rates and pathways of atmospheric oxidation by controlling the budgets of other important atmospheric oxidants (i.e., O_3_, OH, HO_2_, and RO_2_).^4^ NO_*x*_ emissions impact sensitive ecosystems through contributions of excess nitrogen.^5−7^ In the U.S., emission controls have reduced NO_*x*_ emissions from the transportation and power generation sectors by more than half over the last two decades, even as activity has increased.^8,9^ Other parts of the world lag, but the increasing electrification of transportation and of thermal control in buildings, along with the growth of renewable electricity generation, put all countries on a path toward much lower combustion-related NO_*x*_ emissions. It has recently been shown that, as a result of this trend, air quality is increasingly sensitive to emissions of NO from soils,^10^ particularly in agricultural regions where fertilizer application leads to NO_*x*_ soil emissions larger than in many natural ecosystems. In light of this, a better understanding of the role of the biosphere as a source and sink of NO_*x*_ is needed to assess the current and future roles that NO_*x*_ emissions play in affecting human health and ecosystems.

NO and NO_2_ are highly reactive molecules that occur at trace levels (on the order of parts per billion or parts per trillion by volume) in the atmosphere.^11−13^ Rapid photochemical reactions driven by sunlight (*hν*) and other atmospheric oxidants (i.e., O_3_, OH, HO_2_, and RO_2_) interconvert NO and NO_2_ on the time scale of minutes in both urban and remote atmospheres.^4,11,12^ The oxidation of NO_*x*_ leads to the production of nitric acid (HNO_3_), alkyl nitrates (ANs, RONO_2_), and peroxy nitrates (PNs, RO_2_NO_2_) which results in an atmospheric lifetime for NO_*x*_ of ∼5 h.^13−16^ Removal of NO_*x*_ from active chemistry occurs primarily by the first two of these pathways, via either the reaction of OH radicals with NO_2_ to form HNO_3_ or the reaction of NO with RO_2_ radicals to form RONO_2_.^13−20^ RO_2_ is a family of intermediates in the oxidation of organic compounds. On short spatial scales, gas-phase HNO_3_ is nearly chemically inert, with a lifetime to chemical conversion back to NO_*x*_ of ∼50 h. This time scale is long compared to typical time scales for removal by dry deposition (contact removal with surfaces) or wet deposition (dissolution in droplets and subsequent rainout).^21^ RONO_2_ is removed from the atmosphere through reactions that reform NO_*x*_, reactions in aerosols that result in heterogeneous hydrolysis to form HNO_3_, and direct deposition.^14,15,21^ The relative importance of HNO_3_ and RONO_2_ production influences the lifetime of NO_*x*_, the production of O_3_, and the formation of secondary organic aerosol in a wide range of environments.^13−20^ RO_2_ can also react with NO_2_ to form RO_2_NO_2_ at rates similar to that of the formation of RONO_2_.^22^ However, in the lower atmosphere most RO_2_NO_2_ species thermally dissociate to reform NO_2_ on time scales of seconds or minutes, making them a short-lived reservoir of NO_*x*_ with little effect on the overall rate of removal of NO_*x*_ from the atmosphere.^15^ During the night, in the absence of photochemistry, NO_2_ reacts with O_3_ to form NO_3_, which then reacts with NO_2_ to form N_2_O_5_. N_2_O_5_ is highly soluble and is scavenged by aerosols to form HNO_3_ via heterogeneous hydrolysis.^23^ At night, NO_3_ can also react with alkenes to form RONO_2_.^23^ The nitrogen oxides NO_*x*_ along with the oxidation products HNO_3_, ANs, and PNs make up the chemical family NO_*y*_.

**Figure 1:**
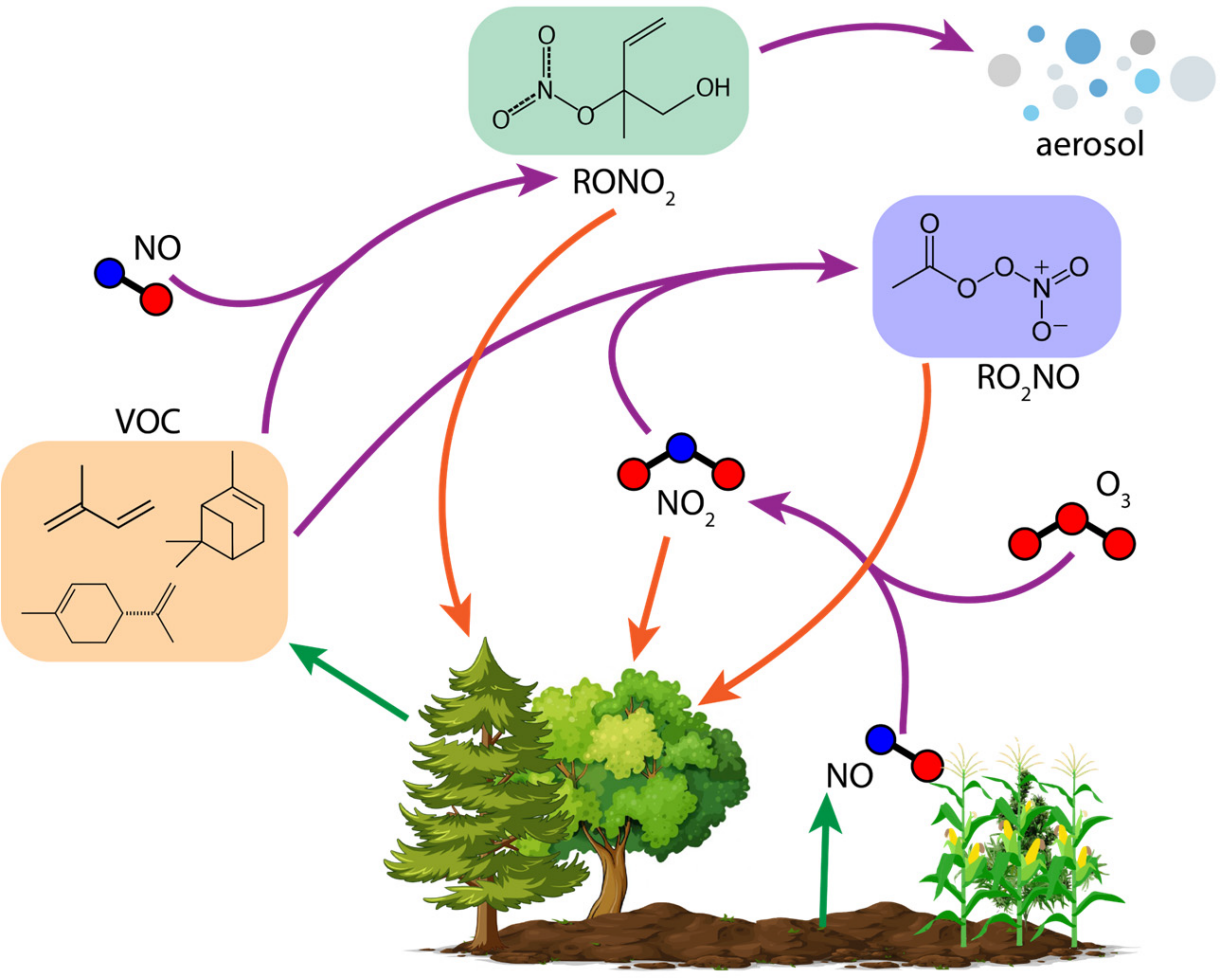
Atmosphere–biosphere exchange processes and chemical transformations of gas-phase atmospherically oxidized nitrogen.

In addition to chemical processes and deposition, plants and microbes can directly affect NO_*x*_. Figure 1 describes these pathways within the atmosphere and the connections to the biosphere. Microbes utilizing N in soils (including natural levels of N and levels perturbed by long-term deposition or enhanced levels from fertilizer application) contribute to emissions of NO into the atmosphere.^24−27^ In a remote forested environment or agricultural region, the primary source of NO_*x*_ in the atmosphere is the emission of NO from soil microbial activity.^24−29^ Soil NO emissions increase with fertilizer applications in agricultural areas^24^ and vary by region, soil type, soil temperature, and a variety of other environmental factors.^25^ Plants can also remove NO_*x*_ from the atmosphere during photosynthesis as a result of chemistry occurring within their stomata.^26,28,29^

Only a fraction of the soil-emitted NO_*x*_ is ventilated into the atmosphere above plant canopies.^26,28,29^ Some of this NO_*x*_ is chemically transformed to alkyl nitrates.^30,31^ The deposition of soil-emitted NO_*x*_ to trees and understory vegetation is also considered to be an important, perhaps dominant, cause of this canopy NO_*x*_ reduction.^29^ Laboratory studies have observed the direct and rapid deposition of NO_*x*_ to plants.^1,2,32−35^ Direct deposition of the RO_2_NO_2_ species peroxyacetyl nitrate (PAN) has also been observed, representing an additional biologically mediated path for the permanent removal of NO_*x*_.^3,36,37^

In this article, we synthesize our understanding of the role of the biosphere as path for the removal of NO_*x*_ from the atmosphere. We review recent laboratory experiments on the rates and mechanisms of removal of atmospheric nitrogen oxides (NO_*y*_) by plants, discuss insights these experiments provide for understanding emissions of NO_*x*_ from soils, and describe a box model that explores these effects in detail. Our working model starts with a standard approximation to the flux of atmospheric gases to surfaces, in this case, a plant with leaves
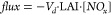
1where LAI is the leaf area index, which is the ratio of the total leaf area in a region to the ground area, and *V*_*d*_ is the deposition velocity.

**Figure 2:**
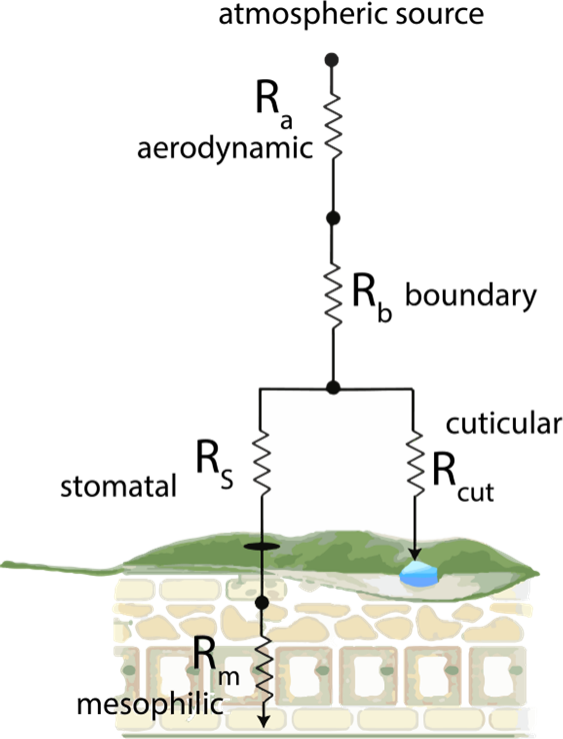
Resistance model for the deposition of an atmospheric trace gas to a leaf.

The deposition velocity is represented using the resistance model framework of Baldocchi et al.^38^ and Wesely et al.^39^ (Figure 2). An atmospheric trace gas, such as NO or NO_2_, must pass through a series of “barriers”, analogous to resistors in an electrical circuit, on its path to removal by deposition. Transfer of the trace gas over each of these “barriers” is modeled as a steady-state process. The first resistor is the aerodynamic resistance (*R*_*a*_), which describes the resistance associated with a trace gas diffusing through a turbulently flowing parcel of air and reaching the surface of a leaf. This parameter is dependent upon the diffusivity of the gas in question and the wind speed. The second resistor is the boundary layer resistance (*R*_*b*_) and represents the diffusion of a trace gas through a region of the laminarly flowing air layer directly above a leaf surface. This parameter is dependent upon microscopic surface properties of the leaf and trace gas diffusivity. Once a trace gas reaches the leaf surface, it can deposit directly onto the cuticles (i.e., leaf surface) (with a resistance represented as *R*_*cut*_) or diffuse through the stomata, which are pores in the leaf through which CO_2_, O_2_, and H_2_O are exchanged. Stomatal behavior is an essential feature of our understanding of molecular exchange during photosynthesis, respiration, and transpiration.^40−43^ The resistance to deposition through the stomata (*R*_*s*_) reflects the rate of physical diffusion through these pores and is the inverse of the stomatal conductance (*g*_*s*_), which represents the degree of stomatal opening. Leaf stomata generally act to maximize CO_2_ uptake while minimizing water loss,^42^ and the resulting *g*_*s*_ is limited by many factors, including light availability, vapor pressure deficit, soil moisture, plant species, leaf age, and season, among others. Once the trace gas enters the stomata, it can undergo hydrolysis and enzymatic reactions within the leaf tissue. This latter step determines the resistance of the mesophyll (*R*_*m*_). If these reactions are relatively rapid, then stomatal deposition is limited primarily by diffusion through the stomata.

A variety of gases deposit onto leaves in this manner, including CO_2_, VOCs, O_3_, H_2_O_2_, HNO_3_, peroxyacyl nitrate, and alkyl nitrates. The exact chemical mechanism for the deposition of many gases is not widely known. The relative contribution of cuticular deposition vs stomatal deposition depends largely on the gas solubility, reactivity of the gases with the leaf surface, and enzymatic reactions within the leaf mesophyll. For example, CO_2_ deposition is entirely stomatal and driven by enzymatic reactions. The deposition of O_3_ includes contributions both from deposition to cuticles and through stomates.^44,45^ Oxidized VOCs (OVOCs) and nitrates may also exhibit both stomata and nonstomatal (cuticular) deposition.^21^ HNO_3_ and H_2_O_2_ are known to proceed at the turbulence rate, indicating that they deposit onto any surface at a rate limited primarily by *R_b_* and *R*_*a*_.^21^

The cuticular deposition of gases, such as OVOCs, O_3_, H_2_O_2_, and HNO_3_, is thought to proceed primarily through dissolution of the gas to aqueous films on the leaf surface, with more rapid cuticular deposition for gases with higher solubility. The rate of cuticular deposition also depends on the reactivity of the gas with the cuticle (particularly for O_3_), which is affected by deposited aerosols, the cuticular wax itself, and compounds secreted by the leaf.^44^ Stomatal deposition is thought to proceed through much the same manner, with trace gases dissolving into the aqueous phase and reacting with compounds within the leaf mesophyll. NO_2_, for example, is thought to form nitrate and nitrite in the aqueous phase of the apoplast and react with nitrate reductase to form ammonium, which then becomes assimilated into amino acids.^46,47^ The uptake of acyl peroxynitrates is likely also facilitated by enzymatic reactions.^3^

The total deposition velocity for trace gas *X* to vegetation, represented by the effects of all pathways shown in Figure 2, can then be represented in eq 2.
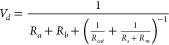
2The parameters in eq 2 are not, in general, known, and describing them for all atmospheric situations is challenging. The simplification introduced by Wesely et al.^39^ has been widely adopted in global chemical transport models. In the Wesely model, stomatal conductance is represented as a function of temperature (*T*/°C) over relevant environmental temperatures and solar radiation (*SR*):
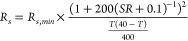
3*R*_*s,min*_ is the minimum stomatal resistance for a given vegetation class. The additional surface resistances (*R*_*m*_ and *R*_*c*_) are functions of the Henry’s law constant (H*) and a reactivity parameter *f*_0_
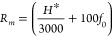
4
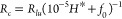
5where a specific value for *R*_*lu*_ is chosen for each ecosystem classification.

The deposition of NO_*x*_ and its oxidation products (particularly RO_2_NO_2_) can proceed at rates that are comparable to the production of HNO_3_ or RONO_2_, thus influencing the concentration of NO_*x*_ in the atmosphere.^48^ This Account focuses on some of our recent research investigating the deposition of NO_*x*_, RONO_2_, and RO_2_NO_2_ and the role of these deposition pathways in controlling NO_*x*_ loss and lifetimes. For the purposes of this Account, we define “deposition” as the physical removal process due to the deposition onto leaf surfaces.

## 2 Flux Measurement Approaches

Observing the exchange flux between the atmosphere and the biosphere is considerably more challenging than collecting concentration measurements. Flux measurements typically occur on the leaf, branch, or ecosystem scale. Fluxes can also be inferred through coupling concentration measurements to chemical transport models. Each approach has its own corresponding set of benefits and difficulties. It is our opinion that a combination of these approaches is necessary for continued advancements in understanding atmosphere–biosphere interactions.

Many approaches exist for measuring the concentrations of relevant gas species. Concentration measurements can be coupled with atmospheric chemical transport models to derive estimates of the biosphere fluxes of NO_*y*_.^24,49,50^ Many atmospheric models have employed a “canopy reduction factor” to account for the loss of NO_*x*_ within the canopy.^24,26,27,50^ However, this parameter is nonphysical and includes loss due to both chemical transformations and deposition, thus misrepresenting the chemical system. Other inferences rely on complicated assumptions for model parametrization, leading to inaccuracies. Nevertheless, concentration measurements, especially those derived from operational networks and satellite observations, provide temporal and spatial coverage that allows for the assessment of effects over multiple years and across multiple ecosystems. Such comparisons are essential to observing sufficient variation in the controlling parameters to test whether our ideas represent the physical process governing the biosphere–atmosphere exchange. Satellites have also shown promise for estimating the rate of some plant physiological processes, such as in our recent reports.^51−53^

Ecosystem-scale flux towers provide a more localized representation than observations from space. Such flux tower observations incorporate differences between species, individual plants, and individual leaves and can be used to infer atmosphere–biosphere exchange rates.^31,54−57^ However, deconvoluting chemistry, deposition, and emission processes beneath a canopy can be challenging. Efforts to do so are confounded by uncertainties in soil NO emission rates and the rates of chemical transformations. This complicates our ability to elucidate the actual deposition fluxes of individual reactive nitrogen oxides from the above-canopy observations and distinguish them from chemical losses. Furthermore, the measurements and flux processes identified at a single ecosystem flux tower site are not necessarily interchangeable with other sites (different soil conditions, sunlight availability, etc., can affect plant physiology). Recent investigations have also shown the potential for aircraft flux measurements of NO_*x*_.^58^ Such advancements in airborne measurements of NO_*x*_ fluxes can allow for the evaluation of fluxes over larger spatial scales and across multiple ecosystems.

Leaf-level flux measurements typically consist of a small controlled environmental chamber enclosing a leaf.^35,59,60^ Branch-level flux measurements are similar but utilize larger chambers that can enclose multiple leaves on a branch.^1−3,32,33,61^ Trace gas exchange in these chambers is calculated by measuring either the concentration difference between the inlet and outlet to a single chamber^1−3,36,35,60^ or the difference between a chamber enclosing a leaf or branch and an empty reference chamber.^32,37,59,61−63^ The latter method is typically used when conducting measurements in the field, as the dual chamber method can simplify accounting for photochemical reactions that can occur upon exposure to ambient UV light. Leaf-level measurements are made easier by the existence of commercially available leaf chambers that provide accurate measurements of stomatal conductance and leaf area (e.g., the Licor-6800 environmental chamber). However, individual leaves can behave differently than the aggregate plant; therefore, leaf chamber measurements typically require repeated measurements over a statistical representation of leaves on a given plant. Fluxes can also be small and close to the limit of detection of the trace gas detector. Branch measurements benefit from larger total exchange rates and are more representative of the aggregate plant, but obtaining accurate measurements of leaf area and stomatal conductance is more challenging. However, research in our group and in other groups has led to approaches that reliably calculate both the enclosed leaf area and the stomatal conductance from measured water vapor fluxes.^1−3,64^ Laboratory experiments have the benefit of being able to quantify the dependence of NO_*x*_ deposition on the temperature, light intensity, humidity, soil water content, and other environmental factors by varying these terms individually.

## 3 Leaf- and Branch-Level Flux Observations

Numerous studies have measured leaf- and branch-level fluxes of NO_*y*_, particularly of NO_2_, NO, and peroxyacetyl nitrate (PAN, C_2_H_3_NO_5_). One study to date has directly measured the deposition of an additional RO_2_NO_2_ species,^3^ and two have investigated the deposition of alkyl nitrates.^60,64^ Here we provide an overview of the findings from leaf- and branch-level NO_*x*_, AN, and PN deposition measurements since the year 2000 and highlight the recent contributions from our group.

### 3.1 NO and NO_2_

**Figure 3:**
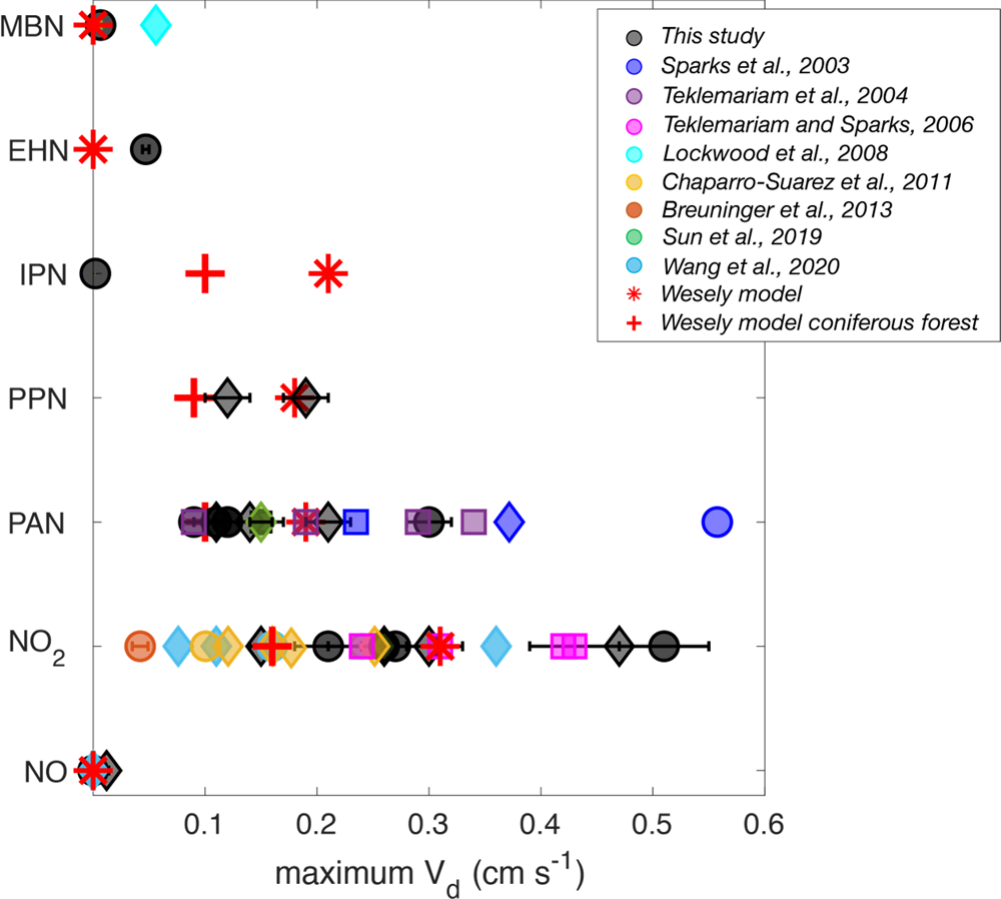
Maximum deposition velocities of NO_2_, peroxyacyl nitrate (PAN), peroxypropionic nitrate (PPN), isopropyl nitrate (IPN), ethylhexyl nitrate (EHN), and methylbutyl nitrate (MBN) as reported in the literature. Different marker colors represent data from different investigations, with data from the three key references (refs (1−3)) shown in black. Error bars associated with black markers are one standard deviation. Each marker represents a different tree species, as detailed in Tables S1–S3. Conifer, broadleaf, and herbaceous species are represented as circles, diamonds, and squares, respectively. Error bars associated with these markers are one standard deviation. Associated error bars are the errors in the measurements when reported. Red crosses represent the maximum leaf-level *V*_*d*_ prescribed by the Wesely model for evergreen forests as implemented in GEOS-Chem. Red asterisks represent the maximum leaf-level *V*_*d*_ prescribed by the Weslely model for all other plant species, as implemented in GEOS-Chem.

Understanding the leaf-level deposition of NO_*x*_ has remained elusive. Experiments have resulted in a wide range of deposition velocities for similar tree species (i.e., evergreen needleleaf, evergreen broadleaf, deciduous broadleaf, etc.) (Figure 3, Table S1). These broad classifications are used in global chemical transport models for representing foliar deposition.^65^ Discrepancies exist in the degree of mesophilic influence on the total uptake rate. Some studies^32^ have found that deposition rates are controlled primarily by stomatal conductance, while others^33,35,61^ have observed lower deposition rates due to substantial additional barriers. Many direct leaf-level laboratory measurements have observed the emission, rather than the deposition, of NO_2_ and NO at the low NO_*x*_ mixing ratios relevant to remote forested environments. This would imply that vegetation acts as a large additional source of NO_*x*_ for the atmosphere, with a source strength on the order of 10^10^ molecules cm^–2^ s^–1^, contrary to field observations suggesting that the biosphere sink of NO_*x*_ is roughly of the same magnitude.^29^ The ambient NO_*x*_ concentration at which vegetation crosses from serving as a sink to a source of NO_*x*_ is known as the compensation point. Compensation points of NO_2_ in laboratory experiments have been sometimes observed^35,59,66−68^ and sometimes not.^1,2,32,33,61^ There has, however, been more agreement in measurements of NO fluxes, with researchers generally finding negligible foliar uptake^1,35,61^ and slight emissions below 1 ppb NO.^1,35^

In Delaria et al.,^2^ we conducted the most comprehensive NO_2_ deposition study to date on three to six individuals of six different conifer and four broadleaf tree species. We used a single laboratory dynamic branch chamber with direct laser-induced fluorescence (LIF) detection of NO_2_ and the simultaneous measurement of the stomatal conductance of the enclosed branch. This study concluded that NO_2_ deposition to 10 tree species scaled directly with stomatal conductance, with minimal contribution from the mesophilic resistance. We found no evidence of NO_2_ emission from any of the tree species examined, in agreement with all other direct NO_2_ flux studies over the past decade (Table S4). We concluded that the existence of an NO_2_ compensation point is improbable and that earlier findings of NO_2_ emission were likely due to experimental detection interference. The findings described in Delaria et al.^2^ also support the small to negligible cuticular deposition observed by most other NO_2_ deposition studies (Table S5), with no significant deposition occurring in the absence of light and/or when *g*_*s*_ was near zero.^32,33,59,61^ We also observed significant nighttime NO_2_ deposition that could be explained by stomates remaining slightly open in the dark, as has been observed in numerous other studies.^69−71^ This effect is not represented by the Wesely model as embedded in CTMs.

**Figure 4:**
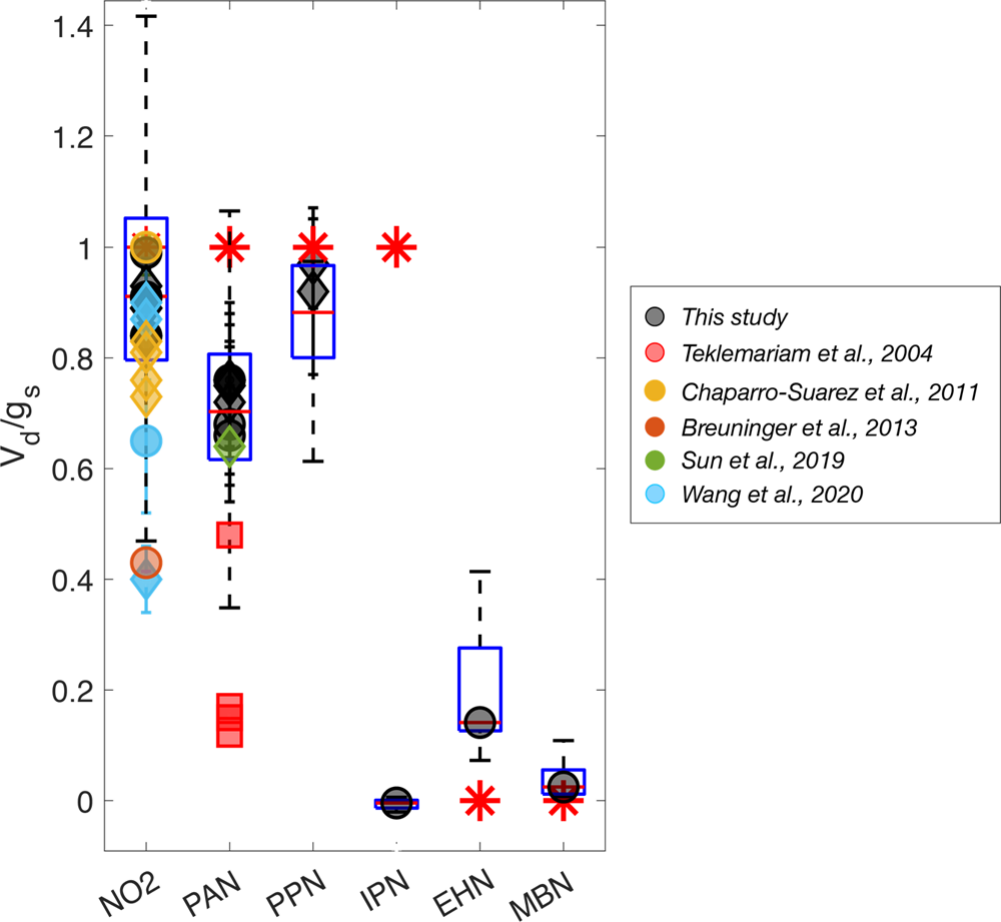
Ratios of the deposition velocity to the stomatal conductance for NO_2_, peroxyacyl nitrate (PAN), peroxypropionic nitrate (PPN), isopropyl nitrate (IPN), ethylhexyl nitrate (EHN), and methylbutyl nitrate (MBN). Box and whiskers represent the aggregated data from all individuals of the different plant species as reported in refs (1−3). Different marker colors represent data from different investigations, with data from the three key references (refs (1−3)) shown in black. Error bars associated with black markers are one standard deviation. Each marker represents the average of data for a different tree species, as detailed in Tables S1–S3. Conifer, broadleaf, and herbaceous species are represented as circles, diamonds, and squares, respectively. V_*d*_/g_*s*_ ratios were calculated from available V_*d*_ and g_*s*_ data and were not explicitly reported. Red asterisks represent the values prescribed by the Wesely model for all vegetation species.

The largest inconsistencies remaining across NO_2_ deposition studies are the degree to which mesophilic processes limit deposition, the range of stomatal conductance *g*_*s*_, and the resultant deposition velocities (Figures 3 and 4, Table S1). The rates of stomatal uptake (*R*_s_ and *R*_m_) depend on both the rate of diffusion through stomata and the rate of reaction within the mesophyll. For species with fast mesophilic reactions (e.g., O_3_), the rate of stomatal deposition is limited primarily by the rate of diffusion through the stomata (*g*_*s*_). In Table S1, we represent the mesophilic effect by the parameter *V*_*d*_/*g*_*s*_, where *g*_*s*_ is the stomatal conductance to the given gaseous species. We have calculated this parameter from stomatal conductance and trace gas flux data presented when it was not explicitly reported (Table S1). Although Delaria et al.^2^ found only minimal mesophilic resistances across the 10 species studied (*V*_*d*_/*g*_*s*_ = 0.79—0.99), Wang et al.^61^ found larger mesophilic effects in the broadleaf deciduous tree *Acer rubrum* and the coniferous species *Pinus strobus*. A range of *V*_*d*_/*g*_*s*_ ratios have been observed from 0.65 (*Pinus strobus*, Wang et al.^61^) to 0.99 (*Pinus contorta*, Delaria et al.^2^ and *Pinus sylvestris*, Chaparro-Suarez et al.^32^) among coniferous species and 0.65 (*Quercus rubra*, Wang et al.^61^) to 0.93 (*Arbutus menziesii*, Delaria et al.^2^) among broadleaf species. Despite the range in reported *V*_*d*_/*g*_*s*_ ratios, the Wesley model for NO_2_ assumes negligible mesophilic resistance, with a reactivity parameter (*f*_0_) of 0.1. It is possible that variations in the reactivity of NO_2_ within the leaf mesophyll between different plant species (i.e., variations in *f*_0_) are partially responsible for the observed differences in *V*_*d*_/*g*_*s*_.

Leaf- and branch-level NO_2_ deposition experiments also observed a wide range of deposition velocities. Different light and humidity conditions among these studies may contribute to the range of observed deposition velocities and stomatal responses. Different tree species have also been found to have different stomatal conductance under similar field conditions, which would clearly play a role in creating the observed disparities between the stomatal deposition rates on different tree species.^72−74^ Observed maximum deposition velocities range from 0.042 cm s^–1^ (*Picea abies*, Breuninger et al.^33^) to 0.51 cm s^–1^ (*Pinus sabiniana*, Delaria et al.^2^) among coniferous trees and 0.11 cm s^–1^ (*Acer rubrum*, Wang et al.^61^) to 0.47 cm s^–1^ (*Acer macrophyllum*, Delaria et al.^2^) among broadleaf trees. Some of the variability in *V*_*d*_*/g*_*s*_ may be explained by this spread in observed deposition velocities, as mesophilic resistances become relatively more important at a larger stomatal conductance. The range of observed deposition velocities reflects a large range of stomatal conductance, likely influenced by species variation and differences in the experimental conditions. This variability in deposition velocities and in the stomatal conductance among species of the same ecosystem class is not represented in most atmospheric CTMs. Modeling the diversity of stomatal responses not only has important implications for representing nitrogen oxide deposition but also for many other species exchanged with stomata, including many biogenic volatile organic compounds, ozone, and CO_2_. Accurate representations of leaf-level exchange necessitate experimental and observational data on the dominant vegetation in an area.

### 3.2 Peroxy Nitrates

Several, but considerably fewer, studies have investigated the deposition of peroxy nitrates to stomata. By far the most studied RO_2_NO_2_ species is peroxy acetyl nitrate (PAN). Like that of NO_2_, a wide variety of deposition velocities have been observed. Teklemariam and Sparks^63^ and Sparks et al.^36^ identified rapid deposition velocities of PAN to herbaceous species of 0.23—0.55 cm s^–1^, but with considerable mesophilic resistances and *V*_*d*_/*g*_*s*_ ratios of 0.12–0.48. (These values were recalculated from those originally reported in these studies to be consistent with models and more recent treatments of deposition velocities.^36,63^) More recent studies have been unable to reproduce these rapid uptake rates. Place et al.^3^ conducted an exhaustive investigation of the uptake rates of 10 tree species and observed deposition velocities ranging from 0.09 cm s^–1^ (*Quercus agrifolia*) to 0.21 cm s^–1^ (*Acer macrophyllum*) for broadleaf species and from 0.11 cm s^–1^ (*Pinus ponderosa, Pseudotsuga menziesii*) to 0.3 cm s^–1^ (*Pinus sabiniana*) for conifer trees. Place et al.^3^ also identified *V*_*d*_/*g*_*s*_ ratios ranging from 0.66 to 0.76. Sun et al.^37^ recently calculated a similar deposition velocity of approximately 0.15 cm s^–1^ and a *V*_*d*_/*g*_*s*_ of 0.64 for a broadleaf tree species (*Quercus ilex*). Although all four of these studies have identified mesophilic limits to the PAN uptake rate, the Wesely model representation predicts negligible mesophilic effects. Additionally, Sun et al.^62^ suggested that over 20% of PAN foliar deposition is nonstomatal, while Place et al.^3^ found no evidence for cuticular PAN deposition. As with NO_2_, all observed nighttime PAN deposition was attributed to nontotal stomatal closure in the latter study. This may indicate a noncuticular component to the nighttime deposition of PAN that has been observed in several field studies (i.e., deposition to other surfaces such as soils or below-canopy chemical loss).^75,76^ More direct uptake experiments on a wider variety of plant species using updated sensitive trace gas detection methods are needed to resolve the rates of potential nonstomatal deposition and allow for more comprehensive treatments of PAN foliar exchange.

Place et al.^3^ was the first investigation of the deposition rate of an additional peroxy nitrate, peroxypropionic nitrate (PPN). We found deposition velocities of 0.19 and 0.12 cm s^–1^ for two different broadleaf tree species—*Acer macrophyllum* and *Quercus douglasii*, respectively—and minimal mesophilic dependence on uptake rates across experimental conditions. Additional experiments should, however, be conducted to test the applicability of these findings to different plant species and to other atmospherically relevant peroxy nitrates.

### 3.3 Alkyl Nitrates

Lockwood et al.^60^ observed the deposition of RONO_2_ to quaking aspen leaves by dosing the tree leaves with high concentrations of methylbutyl nitrate (MBN).^60^ In this study, all methylbutyl nitrate deposition occurred through stomatal uptake with a deposition velocity of 0.056 cm s^–1^, and the mesophilic resistance (*R*_*m*_) was rate-limiting. Order of magnitude slower deposition velocities were identified by Place et al.^64^ for MBN to *Pinus sabiniana* trees. Place et al.^64^ also measured the deposition velocities of 0.0019 and 0.047 cm s^–1^ for isopropyl nitrate (IPN) and ethylhexyl nitrate (EHN), respectively, finding that the mesophilic processing rate was rate-limiting. Clearly, the structure of the *R* functional group changes the deposition behavior.

Nighttime flux tower measurements of C_1_–C_5_ alkyl nitrates in Colorado and New Hampshire revealed moderate nighttime deposition velocities for these compounds.^50,51^ These deposition velocities were attributed to uptake on tree/soil surfaces, assuming that leaf stomata are closed at night. Measurements of multifunctional alkyl nitrates during the Southern Oxidant and Aerosol Study (SOAS) have shown that highly functionalized nitrates deposit rapidly from the atmosphere, some at rates similar to that for nitric acid.^21^ These findings have yet to be validated by controlled leaf-, soil-, and branch-level experiments. Developments in experimental methods are needed to assess the deposition fluxes of a wider variety of RONO_2_ species.

## 4 Implications

**Figure 5:**
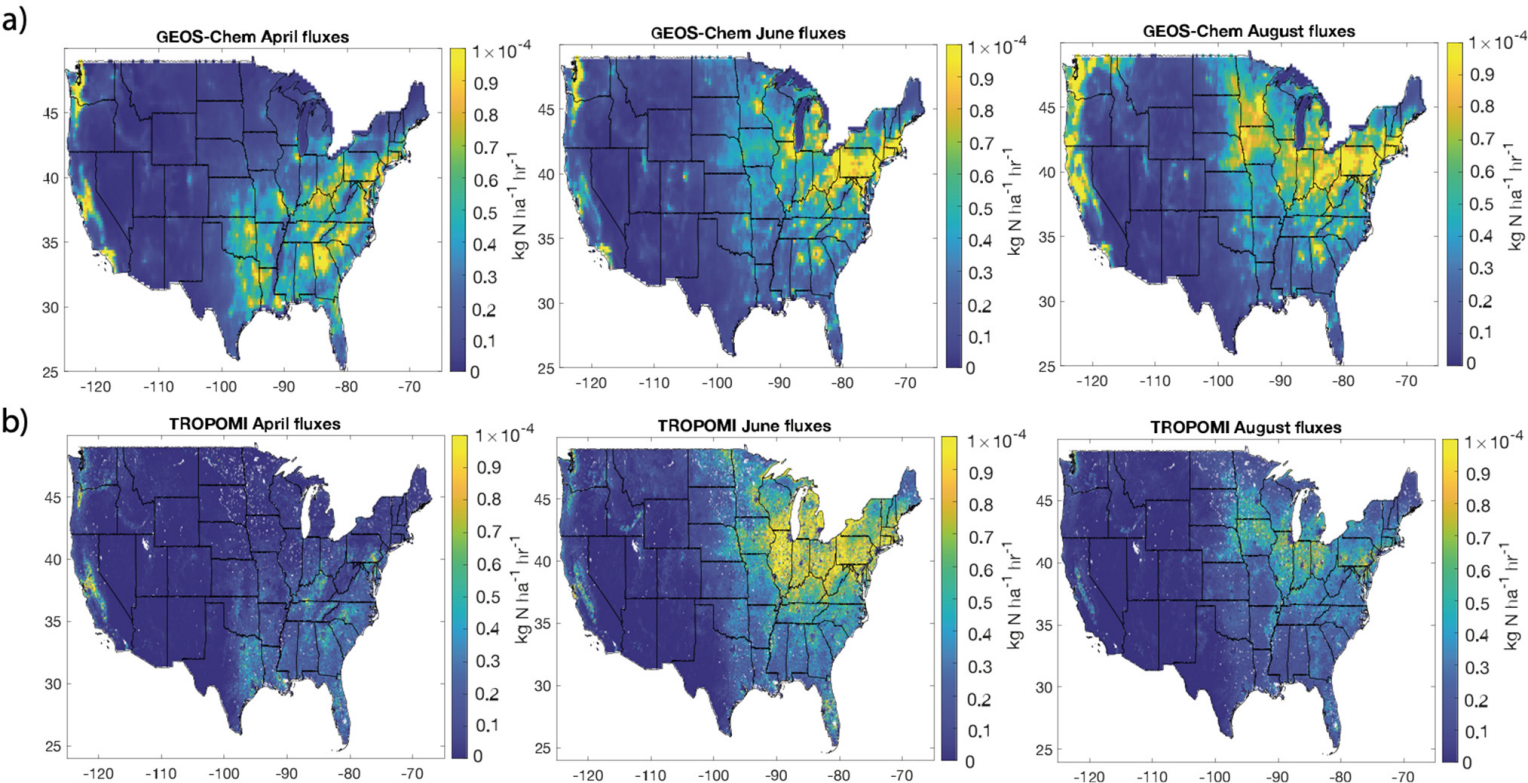
NO_2_ fluxes as calculated from GEOS-Chem using a Wesely deposition scheme or as inferred from TROPOMI NO_2_ and SIF. Adapted with permission from ref (51). Copyright 2021 American Chemical Society.

In Delaria et al.,^51^ we extrapolated the laboratory observations to derive NO_2_ and PAN fluxes over the continental United States from solar-induced fluorescence (SIF) estimations of the aggregated canopy stomatal conductance, using the scaling factors (*V*_*d*_/*g*_*s*_ ratios) determined in Delaria et al.^2^ and Place et al.^3^ Midday NO_2_ fluxes were calculated with SIF and NO_2_ products from the TROPOspheric Monitoring Instrument (TROPOMI) and compared to midday NO_2_ fluxes predicted by GEOS-Chem for April, June, and August. These comparisons reveal inconsistencies in the magnitude and spatial distribution of fluxes during April and August (Figure 5). The oversimplification of deposition parameters in standard model representations likely fails to capture the diversity of plant physiology and results in the misrepresentation of regional variations in NO_2_ and PAN fluxes.

Our findings suggest strong spatial and temporal variations in stomatal-driven NO_*x*_ fluxes. To explore the impact of stomatal NO_*x*_ fluxes on the NO_*x*_ cycle, we constructed a simple model of PAN and NO_2_ stomatal deposition and thermochemical and chemical losses. Chemical losses of PAN and NO_2_ are represented in reactions R1—R3. PAN deposition and chemistry in R1 are treated as representative of all RO_2_NO_2_ for simplicity.
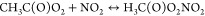
R.1
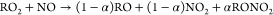
R.2
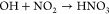
R.3

**Figure 6:**
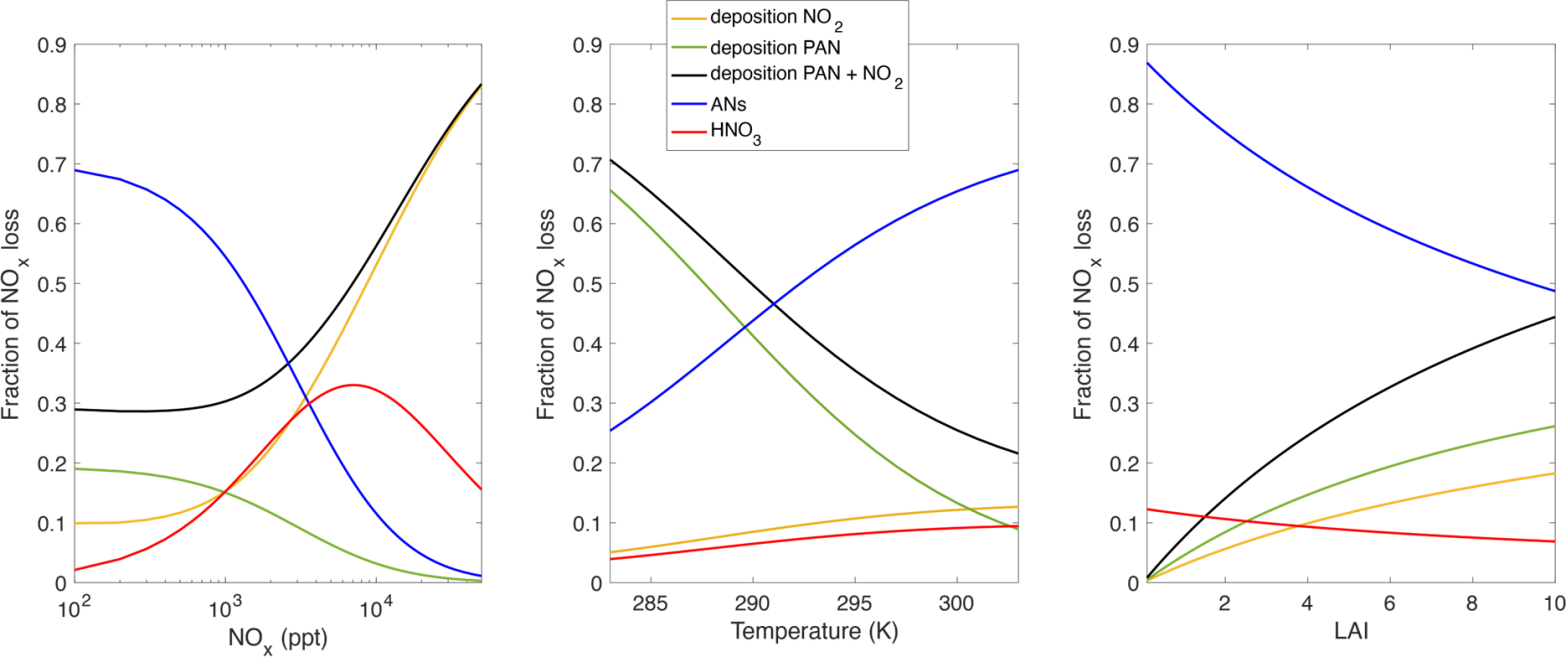
Fraction of total NO_*x*_ that is lost to the deposition for NO_2_, PAN, AN formation, or HNO_3_ formation as a function of (left) the NO_*x*_ mixing ratio, (middle) temperature, and (right) LAI. For this figure, PAN is considered to be an element of NO_*x*_ according to ref (15). For all runs, α = 0.1, VOC reactivity is 8 s^–1^, LAI = 5 m^2^ m^–2^, the HO_*x*_ production rate is 2 × 10^6^ molecules cm^–3^ s^–1^, and the NO_2_ and PAN deposition velocities are the median of what was measured in refs (2) and (3). In the left and right panels, the temperature is set to 298 K, and in the middle and right panels, the NO_*x*_ mixing ratio is 500 ppt.

**Figure 7:**
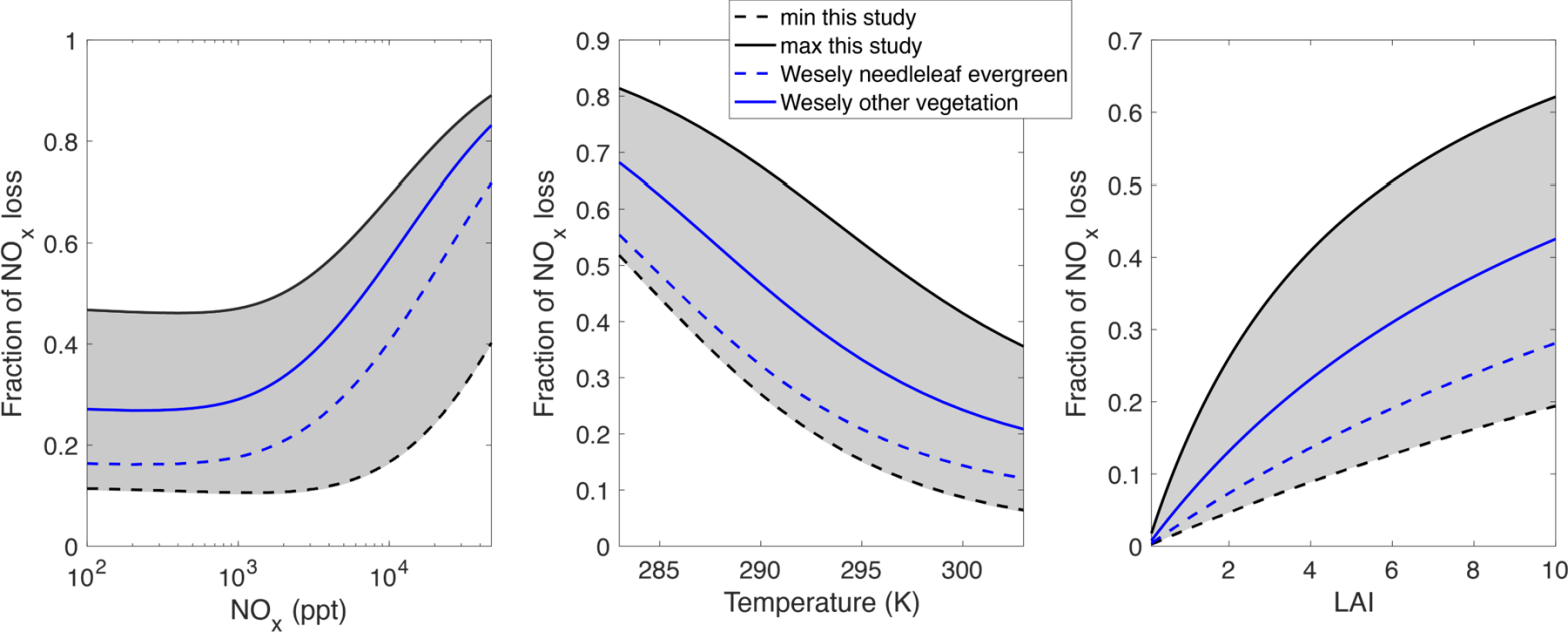
Fraction of total NO_*x*_ that is lost to deposition as a function of (left) the NO_*x*_ mixing ratio, (middle) temperature, and (right) LAI. For this figure, PAN is considered to be an element of NO_*x*_ according to ref (15). Black solid and dashed lines are calculated using the maximum and minimum deposition velocities, respectively, measured in the literature since 2000 (Tables S1 and S2 and Figure 3). Gray shaded regions are the range represented in the literature. Blue solid and dashed lines are calculated using the Wesley model maximum deposition velocity for all nonconifer vegetation and conifer forests, respectively. For all runs, α = 0.1, VOC reactivity is 8 s^–1^, LAI = 5 m^2^ m^–2^, and the HO_*x*_ production rate is 2 × 10^6^ molecules c^–3^ s^–1^. In the left and right panels, the temperature is set to 298 K, and in the middle and right panels, the NO_*x*_ mixing ratio is 500 ppt.

The simple 0D box model is identical to that presented in Perring et al.,^14^ with added PAN chemistry and deposition parameters. The model was set as follows: α = 0.1, VOC reactivity (VOCR) = 8 s^–1^, leaf area index (LAI) = 5 m^2^ m^–2^, and HO_*x*_ production rate (PHO_*x*_) = 2 × 10^6^ molecules cm^–3^ s^–1^. In Figure 6, the NO_2_ and PAN deposition velocities are the median of what we measured and reported in Delaria et al.^2^ and Place et al.^3^ Because it is a short-lived reservoir during the daytime, PAN is included in this model representation as a member of the chemical NO_*x*_ family, as in Romer et al.^15^ We conclude from this simple model that the deposition of PAN and NO_2_ can be responsible for over 20% of the total NO_*x*_ loss at NO_*x*_ mixing ratios of between 100 ppt and 50 ppb and for temperatures of between 10 and 30 °C in forested environments (Figures 6 and 7). This should be taken as an upper limit under these conditions, as the simple model does not consider aerodynamic or boundary resistances. In areas with less-dense foliage (such as in crop fields and grasslands), the NO_*x*_ loss to dry deposition may be considerably lower due to a reduced LAI, while in more dense tropical forests, NO_*x*_ deposition would likely be larger (Figures 6c and 7c). The effect of a lower LAI in certain agricultural and near-urban grasslands would, however, likely also be offset by higher mixing ratios of NO_*x*_ (1—10 ppb) than is typically found in remote forested areas (Figures 6a and 7a).^2,51^ This simple model result is similar to conclusions presented in Delaria et al.,^1^ Delaria and Cohen,^48^ and Place et al.^3^ based on other modeling.

Under the Wesely model as implemented in the current version of GEOS-Chem (v13.3.4), maximal leaf-level deposition velocities, which govern the rate of NO_*x*_ deposition, are prescribed as uniform for almost all plant and ecosystem types, except for conifers, which are treated as a distinct but uniform class. To compare, we applied the box model described above over the range of deposition velocities for PAN and NO_2_ as measured in Place et al.^3^ and Delaria et al.,^2^ respectively. At all NO_*x*_ mixing ratios and temperatures, the fraction of NO_*x*_ loss due to deposition (rather than due to chemical formation of ANs and HNO_3_) is high, with median values in the range of 20–60% (Figure 7). Any value in the observed range indicates an important role for NO_*x*_ deposition as a pathway to removal from the atmosphere. The leaf- and branch-level flux observations of reactive nitrogen oxide deposition collectively emphasize the role of dry deposition in influencing chemical lifetimes and loss pathways.

## 5 Concluding Remarks

Above-canopy NO_*x*_ fluxes represent the sum of all sources and sinks, including soil emission, below-canopy chemistry, and foliar deposition. Our laboratory experiments confirm the essential role of stomatal deposition in the removal of NO_*x*_ from the atmosphere. Over a wide range of relevant atmospheric conditions, we show that the uptake of stomata is responsible for 20–60% of total NO_*x*_ loss in forests and agricultural regions. This uptake renders the deployment of the ad hoc canopy reduction factor, used to limit soil N emissions from reaching the atmosphere, an incomplete representation of canopy reduction. Canopy reduction factors inherently do not treat the atmosphere explicitly, making model-measurement comparisons used to interpret atmospheric processes incongruous. Explicit calculations of soil emission losses using deposition velocities and in-canopy chemistry should be favored over canopy reduction factors in most modeling schemes, given that the necessary parameters for doing so (e.g., stomatal conductance) are already estimated. Canopy reduction factors may be appropriate only in instances where they can be explicitly estimated using simplified representations of chemistry and direct deposition that is specific to the vegetation in a particular region.

Accurate representations of the processes contributing to net canopy fluxes are required to interpret spatial and temporal variations in canopy NO_*x*_ fluxes as well as to understand the biosphere’s contribution to ambient NO_*x*_ mixing ratios. To represent the soil–plant–atmosphere system more accurately, we recommend updating global CTMs to reflect our improved understanding of foliar processes. In particular, parameter *R*_*m*_ in the resistance framework should reflect laboratory findings on the role of the mesophyll in reactive nitrogen foliar uptake. The representation of stomatal conductance should also reflect the observed diversity of plant physiology. Semiempirical dry deposition schemes that couple stomatal conductance with net photosynthesis have shown some promise, particularly when tuned with ecosystem-scale measurements, as discussed in several recent works.^45,77,78^

Due to the importance of soil NO to regional air quality, ozone, and aerosol production, inconsistencies in the treatment of nitrogen fluxes should be addressed. As combustion related NO_*x*_ emissions are reduced, the role of the biosphere as a control over atmospheric NO_*x*_ has increased importance for the chemistry of the atmosphere.

## Supporting Information Available

Summary of oxidized nitrogen deposition experiments, compensation points for NO_2_ emission, and summary of cuticular deposition findings (PDF)
