# Supplementary material for: Measurements of Atmosphere–Biosphere Exchange of Oxidized Nitrogen and Implications for the Chemistry of Atmospheric NOx
Source: Acc Chem Res. 2023 Jun 22;56(13):1720–30. doi: 10.1021/acs.accounts.3c00090 (PMC10324316; doi:10.1021/acs.accounts.3c00090)
Supplement: Supplementary file 1 — ar3c00090_si_001.pdf [file ar3c00090_si_001.pdf]

Supporting Information: Measurements of atmosphere-biosphere exchange of oxidized nitrogen and implications for the chemistry of atmospheric NOx

Erin R. Delaria†,‡ and Ronald C. Cohen,¶,§*

†Atmospheric Chemistry and Dynamics Laboratory, NASA Goddard Space Flight Center, Greenbelt, MD, 20771, USA

‡ Oak Ridge Associated Universities, Oak Ridge, TN, 37830, USA

¶Department of Chemistry, University of California, Berkeley, Berkeley, CA, 94720, USA

§Department of Earth and Planetary Science, University of California, Berkeley, Berkeley, CA, 94720, USA

Table S1: Summary of NO_x deposition experiments

Study	gas	tree species	max Vd (cm/s)	Vd/gs	experiment	detection method	PPFD (umol m^{-2} s^{-1})	RH%
Delaria et al., 2018	NO	*Quercus agrifolia*	0.012	NA	single laboratory branch	NO2 LIF	1190	60
Delaria et al., 2020	NO_2	*Quercus agrifolia*	0.015	0.89	single laboratory branch	NO2 LIF	1190	30-90
		Quercus douglasii	0.30	0.89				
		Acer macrophyllum	0.47	0.84				
		Arbutus menziesii	0.26	0.93				
		Pinus contorta	0.24	0.99				
		Pinus sabiniana	0.51	0.79				
		Pinus ponderosa	0.26	0.91				
		Calocedrus decurrens	0.21	0.91				
		Sequoia sempervirens	0.27	0.84				
		Pseudotsuga menziesii	0.26	0.91				
Wang et al., 2020	NO_2	*Quercus rubra*	0.76	0.87	dual field branch	NO-CL photolytic converter	1086	61
		Acer rubrum	0.11	0.4			1200	72
		Populus grandidenta	0.36	0.90			601	71
		Pinus strobus	0.16	0.65			850	67
	NO	*Quercus rubra*	0	0			1086	61
		Acer rubrum	0	0			1200	72
		Pinus strobus	0	0			601	71
		Populus grandidenta	0	0			850	67
Breuninger et al., 2013	NO_2	*Picea abies*	0.042[a]	~0.4[a]	dual field branch	NO-CL photolytic	319±365	80±14
Chaparro-Suarez et al., 2011	NO_2	*Quercus Ilex*	0.12[b]	0.81[b]	dual laboratory branch	NO-CL photolytic	450 and 900	60
		Quercus robur	0.16[b]	0.83[b]				
		Fagus sylvatica	0.18[b]	0.73[b]				
		Betula pendula	0.25[b]	0.76[b]				
		Pinus sylvestris	0.1[b]	0.99[b]				
Teklemariam and Sparks, 2006	NO_2	*Zea mays*	0.24[c]	ND[d]	Single laboratory leaf	NO-CL photolytic	500-2000	NR[e]
		Helianthus annus	0.42[c]	ND[d]				
		Triticum aestivum	0.43[c]	ND[d]				
		Catharanthus roseus	0.31[c]	ND[d]				
Hereid and Monson, 2001	NO_2	*Zea Mays*	0.19	0.50	Single field leaf	NO-CL luminol	1600	45
	NO		~0.02	<0.1				
Gessler et al., 2002	NO_2	*Picea abies*	~0.16	>1	Dual field branch	NO-CL photolytic	174[f]	60
Gessler et al., 2000	NO_2	*Fagus sylvatica*	0.2±0.1	~1			0—900[f]	30

a. Calculated from reference Fig. 6
b. Calculated from reference Fig. 7
c. Calculated from reference Fig. 3
d. Unable to determine based on information reported in reference.
e. Information was not reported.
f. Photosynthetic photon fluence rate (PPFR) reported instead of PPFD.

Table S2: Summary of PN deposition experiments

Study	gas	tree species	max Vd (cm/s)	Vd/gs	experiment	detection method	PPFD (umol $m^{-2}s^{-1}$)	RH%
Place et al., 2020	PAN	*Quercus agrifolia*	0.09	0.72	single laboratory branch	TD-LIF	1190	30-80
		Quercus douglasii	0.14	0.75				
		Acer macrophyllum	0.21	0.75				
		Arbutus menziesii	0.15	0.76				
		Pinus contorta	0.12	0.76				
		Pinus sabiniana	0.3	0.66				
		Pinus ponderosa	0.11	0.76				
		Calocedrus decurrens	0.12	0.66				
		Sequoia sempervirens	0.15	0.68				
		Pseudotsuga menziesii	0.11	0.66				
	PPN	*Quercus douglasii*	0.12	0.97				
		Acer macrophyllum	0.19	0.92				
Sun et al., [illegible]	PAN	*Quercus Ilex*	0.15	0.64	Dual [illegible]	GC-ECD	600	40 -90
Teklemariam et al., 2004	PAN	*Zea mays*	0.09	0.48	Dual laboratory branch	GC-ECD	1000	30-90
		Helianthus annus	0.29	0.17				
		Triticum aestivum	0.19	0.12				
		Catharanthus roseus	0.34	0.15				
Sparks et al., 2003	PAN	*Zea mays*	0.23	ND[a]	single laboratory branch	GC-ECD	~1000	65
		Quercus velutina	0.37	ND[a]				
		Pinus contorta	0.55	ND[a]				

a. Unable to determine based on information reported in reference.

Table S3: Summary of AN deposition experiments

Study	gas	tree species	max Vd	Vd/gs	experiment	detection method	PPFD (umol m^{-2}	RH%
Place et al., 2021	MBN<br>IPN<br>EHN	*Pinus sabiniana*	0.0066<br>0.0019<br>0.047	0.02<br>0.00<br>0.14	single laboratory branch	TD-LIF	1190	30-80
Lockwood et al., 2008	MBN	*Populus tremuloidus*	0.056	ND[a]	Single laboratory [illegible]	GC	1000	60

a. Unable to determine based on information reported in reference

Table S4: Compensation point for NO_2 emission

Study	tree species	Compensation point (ppt)
Delaria et al., 2020	*Quercus agrifolia*	not significant
	Quercus douglasii	not significant
	Acer macrophyllum	not significant
	Arbutus menziesii	not significant
	Pinus contorta	not significant
	Pinus sabiniana	not significant
	Pinus ponderosa	not significant
	Calocedrus decurrens	20 ± 20[b]
	Sequoia sempervirens	not significant
	Pseudotsuga menziesii	not significant
Wang et al., 2020	*Quercus rubra*	19 ± 56[a]
	Acer rubrum Populus	60 ± 119[a]
	grandidentaPinus	38 ± 59[a]
	strobus	4 ± 60[a]
Breuninger et al., 2013	*Picea abies*	170 ± 150–650 ± 370[a]
Chaparro-Suarez et al., 2011	*Quercus Ilex*	not significant
	Quercus robur	not significant
	Fagus sylvatica	not significant
	Betula pendula	not significant
	Pinus sylvestris	not significant
Teklemariam and Sparks, 2006	*Zea mays*	~1700
	Helianthus annus	~1700
	Triticum aestivum	~1700
	Catharanthus roseus	~1700
Gessler et al., 2002	*Picea abies*	1700
Sparks et al., 2001	*Brosimum utile*	520
	Ficus nympholia	850
	Nectandra pupurescens	1090
	Virola novi	1230
	Manilkara bidentata	1600
Hereid and Monson, 2001	*Zea Mays*	900
Gessler et al., 2000	*Fagus sylvatica*	1800-1900

a. Data determined to be not statistically significant.

b. Data below the instrument limit of detection.

Table S5: Summary of cuticular deposition findings

Study	gas	tree species	V_d cuticular (cm s^{-1})
Delaria et al., 2020		*Quercus agrifolia*	≈0.001
		Quercus douglasii	≈0.001
		Acer macrophyllum	≈0.001
		Arbutus menziesii	≈0.001
		Pinus contorta	≈0.001
		Pinus sabiniana	≈0.001
		Pinus ponderosa	≈0.001
		Calocedrus decurrens	≈0.001
		Sequoia sempervirens	≈0.001
		Pseudotsuga menziesii	≈0.001
Place et al., 2020	PAN	*Quercus agrifolia*	ns[a]
		Quercus douglasii	ns
		Acer macrophyllum	ns
		Arbutus menziesii	ns
		Pinus contorta	ns
		Pinus sabiniana	ns
		Pinus ponderosa	ns
		Calocedrus decurrens	ns
		Sequoia sempervirens	ns
		Pseudotsuga menziesii	ns
	PPN	*Quercus douglasii*	ns
		Acer macrophyllum	ns
Place et al., 2021	MBN	*Pinus sabiniana*	ns
	IPN		ns
	EHN		ns
Wang et al., 2020	NO_2	*Quercus rubra*	0.003±0.002
		Acer rubrum	0.008 ±0.004
		Populus grandidenta	0.006 ±0.005
		Pinus strobus	0.043 ±0.009
Breuninger et al., 2013		*Picea abies*	0.007 ±0.006 , 0.0002 ±0.004[b]
Chaparro-Suarez et al.,2011	NO_2	*Quercus Ilex*	ns
		Quercus robur	ns
		Fagus sylvatica	ns
		Betula pendula	ns
		Pinus sylvestris	ns
Teklemariam and Sparks, 2006	NO_2	*Zea mays*	ND[e]
		Helianthus annus	ND[e]
		Triticum aestivum	ND[e]
		Catharanthus roseus	ND[e]
Gessler et al., 2002	NO_2	*Picea abies*	≈0.006[c]
Gessler et al., 2000	NO_2	*Fagus sylvatica*	ns
Sun et al., 2016b	PAN	*Quercus Ilex*	≈0.01[d]
Sparks et al., 2003	PAN	*Zea mays*	ns
		Quercus velutina	ns
		Pinus contorta	ns

a. not significant
b. Reported for two different trees at g_{H2O} = 0.004s cm s^{-1}.
c. Reported as up to 2 times g_S. Calculated from reference Fig. 4.
d. Reported as 0.21—0.23 of the total deposition flux during the day where F_{PAN} = 8—12 pmol $m^{-2}s^{-1}$ at 574 ppt PAN.
e. Unable to determine a value based on the information reported in reference. Non-zero deposition was observed during stomatal closure, indicating cuticular deposition.
